# Familial hypercholesterolemia with bilateral cholesterol granuloma: A case series

**DOI:** 10.1016/j.ijscr.2019.07.018

**Published:** 2019-07-19

**Authors:** Nouf Albakheet, Yazeed Al-shawi, Mohammed Bafaqeeh, Hanadi Fatani, Yasser Orz, Ibrahim Shami

**Affiliations:** a King Saud bin Abdulaziz University for Health Sciences, P.O. Box: 13247, Riyadh, Saudi Arabia; b King Abdullah Ear Specialist Center, Riyadh, Saudi Arabia; c Prince Sultan Military Hospital. P.O. Box: 12233, Riyadh, Saudi Arabia; d National Neuroscience Institute, P.O. Box: 59046, Riyadh 11525, Saudi Arabia; e KFMC, P.O. Box: 59046, Riyadh 11525, Saudi Arabia; f Adult Neurosurgery, National Neuroscience Institute, Adult Neurosurgery, P.O.BOX: 59046, Riyadh 11525, Saudi Arabia; g Otorhinolaryngology and Head & Neck Surgery Department, Main Hospital, King Fahad Medical City, P.O. Box. 59046, Riyadh 11525, Saudi Arabia

**Keywords:** Cholesterol granuloma, Mastoid cavity, Familial hypercholesterolemia

## Abstract

**Introduction:**

Cholesterol granuloma is a benign mass that commonly involves the petrous apex but rarely affects other structures, such as the mastoid cavity. It is diagnosed histologically by the presence of giant cells, and Its management is individualized based on some factors such as the size and location of the lesion.

**Presentation of case:**

The first case was a 33-year-old man who presented to the outpatient clinic with a two-year history of right-sided pulsatile tinnitus, hearing loss, and vertigo. Upon investigations, a large, destructive mass in the tympanomastoid region was found and managed surgically. The other case was for a 41-year-old man who presented to the emergency department with loss of consciousness. Urgent CT was done and revealed an aggressive hypodense posterior fossa mass destroying the right temporal bone that was managed surgically.

**Discussion:**

In this report, we describe two patients with familial hypercholesterolemia who developed bilateral cholesterol granuloma that were managed surgically.

**Conclusion:**

These cases are reported because of their rare location and presentation since few cases of bilateral cholesterol granuloma have been reported in the literature.

## Highlights

• Sensorineural hearing loss as a consequence of cholesterol granuloma.
• CT scan and MRI are conclusive for the evaluation of cholesterol granuloma lesions.
• Removal of cholesterol granuloma by suctioning and curettage.

## 1 Introduction

This case has been reported in line with PROCESS criteria [1]. Cholesterol granuloma is a slow-growing benign mass with a clinical presentation that varies depending on its location and dimensions. It is most commonly found in the petrous apex of the temporal bone [2]. This report describes two patients with familial hypercholesterolemia and bilateral cholesterol granuloma.

## 2 Case 1

**Fig. 1:**
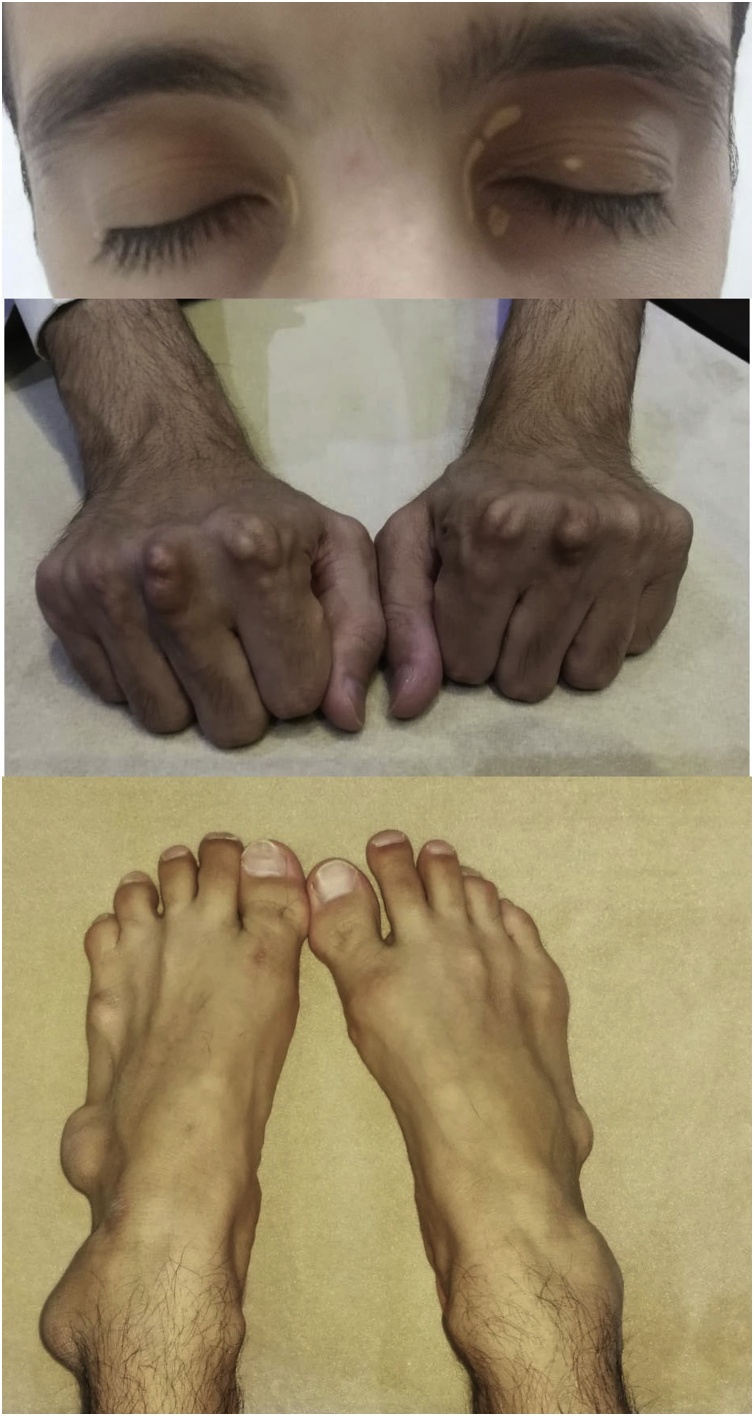
Xanthelasma around both eyelids and multiple xanthomas around joints of both hands and feet.

**Fig. 2:**
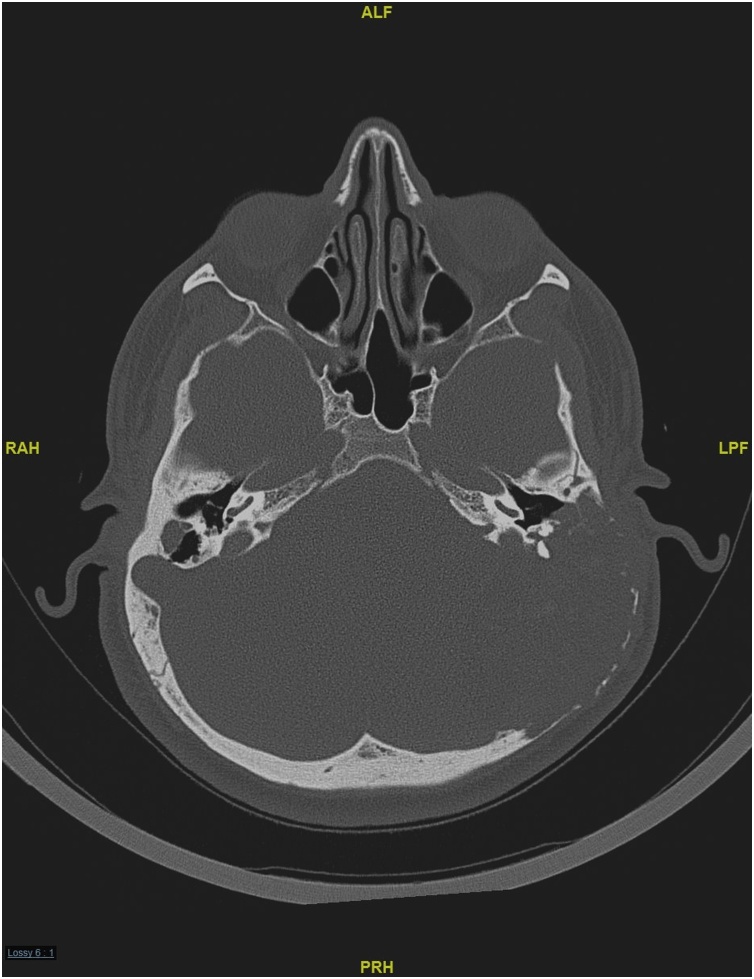
Computed tomography (CT) scan of the temporal bone showing a large, expansile, heterogenous mass, centered in the left tympanomastoid region. The mass was bulging into the external auditory canal, middle ear cavity, and extending posteriorly to temporal and occipital region. Smaller lesion is seen on the right side within the mastoid cavity.

**Fig. 3:**
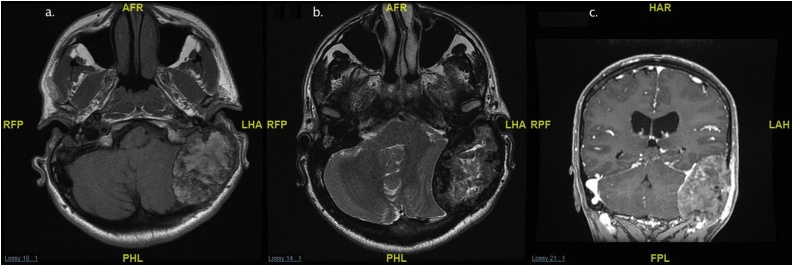
a. T1 without IV contrast. b. T2 weight image. c. Coronal T1 weighted image with IV contrast. Magnetic resonance imaging (MRI) of the temporal bone identified a large, expansile, heterogenous mass in the left tympanomastoid region with involvement of the mastoid air cells, and occipital bone and a mass effect on the cerebellum and left posterior temporal and occipital lobes. The mass showed very tiny foci of enhancement (c) and had intrinsic high-signal intensity on T1 and T2-weighted imaging. A similarly limited but smaller mass was found on the right side.

A 33-year-old man with a diagnosis of familial hypercholesterolemia treated by simvastatin presented to the outpatient clinic in the otolaryngology department of a tertiary hospital with a two-year history of right-sided pulsatile tinnitus, hearing loss, and vertigo. The patient had no history of ear pain or discharge in the right ear and reported no complaints in the left ear. The patient denied any previous surgery or trauma to either ear. His family history was positive for familial hypercholesterolemia, with three first-degree relatives affected. Physical examination revealed multiple xanthomas around the joints in both hands and feet, and there are bilateral eyelid xnthelasma (Fig. 1). Cranial nerves and otoscopic examinations were normal bilaterally. The Rinne test was positive bilaterally and the Weber test was lateralized to the left side. Pure tone audiometry confirmed moderate sensorineural hearing loss with a pure tone average of 40 dB on the right side and hearing thresholds in the normal range on the left side. The speech recognition threshold levels were 40 dB in the right ear and 15 dB in the left ear. A tympanogram showed a type A graph bilaterally. The patient’s laboratory results included a fasting plasma cholesterol level of 17.65 mmol/L (reference range 3.63–5.15) and a low-density lipoprotein cholesterol level of 17.60 mmol/L (reference range 0.00–3.40). A computed tomography (CT) scan of the temporal bone revealed an incidental finding of a large, destructive, heterogenous mass lesion measuring 5.5 cm × 4 cm × 4.2 cm that was centered in the left tympanomastoid region. The mass was bulging into the external auditory canal and middle ear cavity, with a mass effect in the cerebellum causing tonsillar herniation. There was also a smaller lesion of the same appearance and density on the right mastoid measuring 1 cm × 0.75 cm (Fig. 2). Magnetic resonance imaging (MRI) of the temporal bone identified a large, heterogenous mass in the left tympanomastoid region with involvement of the mastoid air cells, occipital bone, and a mass effect on the cerebellum and left posterior temporal and occipital lobes that was causing crowding of the posterior fossa structures and tonsillar herniation. The mass showed very tiny foci of enhancement and had intrinsic high-signal intensity on T1 (Fig. 3a, c) and T2-weighted imaging (Fig. 3b). A similarly limited but smaller mass was found on the right mastoid. There was no intracranial invasion on the right side. A treatment plan was devised that included exploration and biopsy of the left mastoid lesion. However, management of the right ear consisted of observation only because the mass was small and asymptomatic. When sent to preoperative anesthesia clinic, the patient was found to have severe narrowing in several major vessels; therefore, interventions for cholesterol granuloma were delayed because of the high risk of cardiac events during non-cardiac surgery. The patient was referred to cardiology. One year later, he underwent successful coronary artery bypass graft surgery. Eight months later, the patient was admitted to hospital for resection of left ear mass. Intraoperatively, the patient underwent subtotal petrosectomy and the intracranial part of the mass was approached through retrolabyrinthine, retrosegmoid approach. Dissection was carried out in extradural plane. The yellowish to brownish semisolid mass which was destroying the squamous, mastoid and occipital bones, and it was found to be adherent to the middle and posterior fossa dura and extending to the retrofacial air cells and jugular foramen. The mass was resected completely. The external auditory canal was closed using blind sac procedure, the cavity was filled with abdominal fat, and the bone defect was reconstructed using titanium mesh with insertion of an external ventricular drain that was removed after two days. The patient’s postoperative course was uneventful, and he was discharged home on the fifth postoperative day.

**Figs. 4 and 5:**
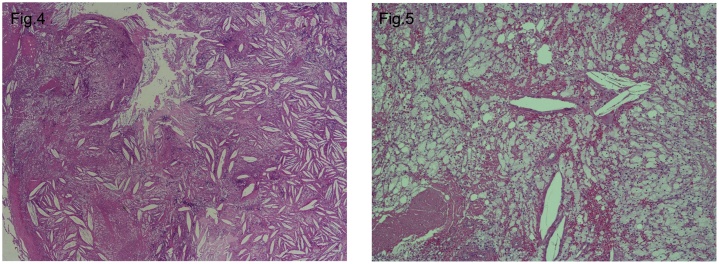
Cholesterol granuloma stained with hematoxylin and eosin.

**Fig. 6:**
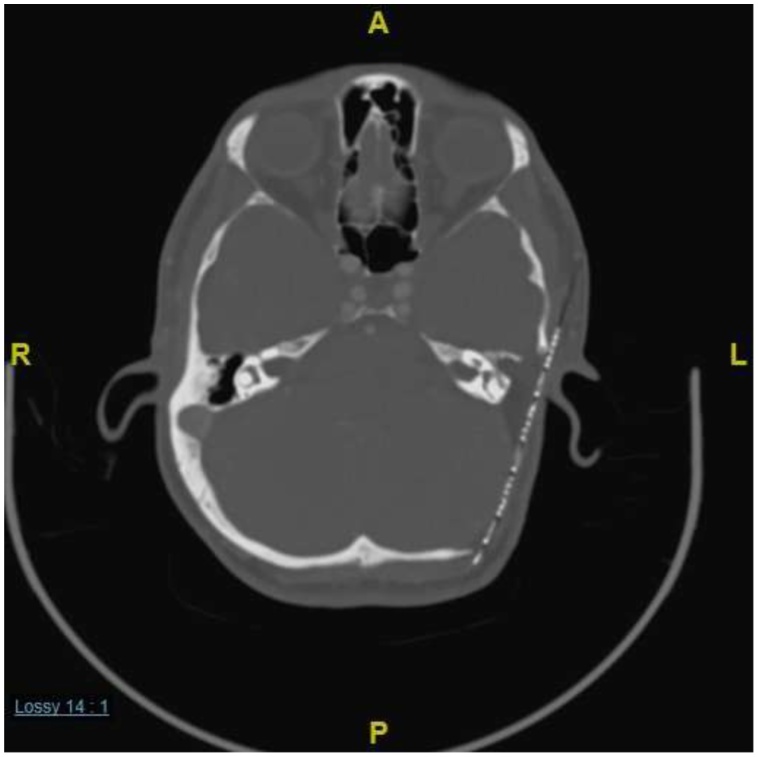
CT axial cut post resection of left temporal cholesterol granuloma.

Histopathological evaluation with hematoxylin and eosin staining confirmed the presence of a destructive infiltrate containing foamy macrophages, foreign body giant cells, and cholesterol clefts (Figs. 4 and 5). On review in the outpatient clinic 6 months later, the patient had no complaints, examination was unremarkable, and radiological imaging confirmed complete resection of the heterogeneous mass with no signs of recurrence (Fig. 6). The mass on the contralateral side showed no signs of progression. The patient continues to be followed up regularly in clinic; his hypercholesterolemia remains controlled and there are no signs of recurrence. Plasma electrophoresis has been planned for further management for his familial hypercholesterolemia.

## 3 Case 2

**Fig. 7:**
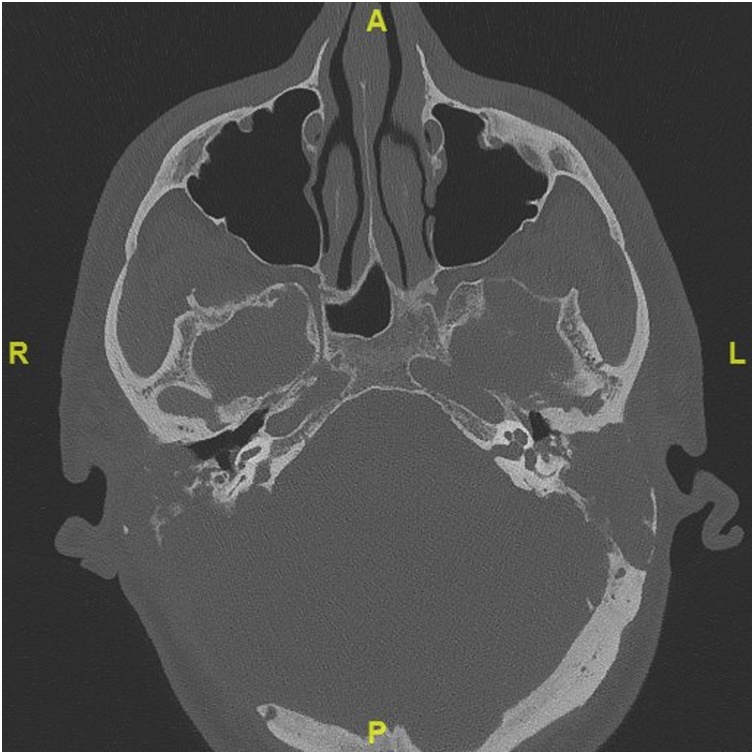
CT scan hypodense, soft tissue, posterior fossa mass destroying the right petrous bone and the adjacent occipital and temporal bones. A similar lesion involving the left mastoid portion of the petrous bone with a small tissue component was seen in the posterior fossa on the left side.

**Fig. 8:**
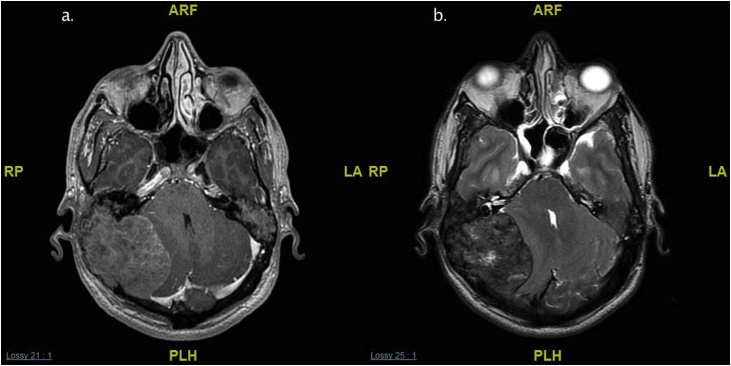
a. T1 weighted image with IV contrast. b. T2 weighted image. MRI showed bilateral heterogenous enhancement of extra-axial posterior fossa lesions originating from the mastoid cavity and petrous bones.

A 41-year-old man with known type 2 diabetes, hypertension, secondary adrenal insufficiency, and homozygous familial dyslipidemia was brought to the emergency department with sudden loss of consciousness. He had had no complaints prior to this episode. His chronic conditions were managed by hydrocortisone, aspirin, lisinopril, hydrochlorothiazide, warfarin, allopurinol, and ezetimibe. His past surgical history was positive for coronary artery bypass surgery and aortic valve replacement at the age of 36 years. His family history included three first-degree relatives with familial dyslipidemia and one first-degree relative with premature cardiac disease. On neurological examination, the patient was disorientated but obeyed commands and opened his eyes spontaneously. No cranial nerve deficit was detected except for hearing loss in the right ear. Pure tone audiometry performed after resolution of the acute condition showed normal ranges in both ears. However, the patient’s laboratory results included a fasting plasma cholesterol level of 18.40 mmol/L and a low-density lipoprotein cholesterol level of 15.4 mmol/L. An urgent CT scan of the head performed in the emergency room showed an aggressive hypodense posterior fossa that was soft tissue mass destroying the right temporal bone and the adjacent occipital bone. The mass measured 45 mm × 65 mm, extended upwards to the posterior temporal bone, and was causing compression and deviation of the fourth ventricle and brain stem with obstructive hydrocephalus. A similarly obstructive lesion involving the mastoid portion of the petrous bone with a small tissue component was seen in the posterior fossa on the left side (Fig. 7). MRI showed bilateral heterogenous enhancement of extra-axial posterior fossa lesions originating from the mastoid cavity and petrous bones. The lesion on the right measured 53 mm × 77 mm × 75 mm and the lesion on the left measured 41 mm × 34 mm × 31 mm (Fig. 8). The patient was taken immediately to the operating room for decompression craniotomy to stabilize his condition. A right retrosegmoid craniotomy was performed intraoperatively. The bone was found to be very thin, and a greasy-looking yellow lesion was found extramurally that was removed easily by suctioning and curettage. Histopathologic examination of the mass confirmed cholesterol granuloma. The patient’s postoperative course was uneventful, and his preoperative complaints improved. The patient was discharged home with a good neurological status. The mass on the left side is being followed up in the otolaryngology and neurosurgery departments and seems to be stable in size.

## 4 Discussion

Cholesterol granuloma was first described by Manassé in 1917, but it was not until 40 decades later that Birrell (1956), Sheehy and McKibbem (1956), and Friedmann (1959) refocused attention on this benign mass [3]. The pathogenesis of cholesterol granuloma is thought to include local hemorrhage as a result of recurrent inflammation, poor ventilation, and lack of drainage of the middle ear cavity. The presentation of cholesterol granuloma differs according to its location and extent. It may be diagnosed incidentally or when a patient presents with conductive hearing loss when the lesion occupies the middle ear. If the lesion is in the petrous apex, the patient could present with sensorineural hearing loss, tinnitus, vertigo, headache, cranial nerve palsy, facial pain, facial numbness, cerebrospinal fluid leak, and/or diplopia. Cholesterol granuloma is being recognized more often nowadays than in the past, probably because of better diagnostic approaches than an increased incidence [2]. CT and MRI are very important in the diagnosis of cholesterol granuloma. On CT, cholesterol granuloma appears as a well-circumscribed expansile lesion that is isodense with the brain and non-contrast enhancing. A CT scan can also show the extent of the lesion. Meanwhile, on MRI, cholesterol granuloma appears as a hyperintense lesion on T1-weighted and T2-weighted images, which reliably differentiates this lesion from cholesteatoma and allows for appropriate planning of a less invasive surgical procedure. Macroscopically, cholesterol granuloma has a shiny appearance and is brownish or yellowish in color. Microscopically, cholesterol granuloma consists of chronic inflammatory granulation tissue containing cholesterol crystals surrounded by giant cells [4]. Treatment for cholesterol granuloma is individualized according to the site, size, and extent of the lesion and whether it shows aggressive or non-aggressive features. Milder non-aggressive and non-symptomatic cases can be followed up with regular MRI scans, but others might require surgical resection. Complete eradication might be not possible. In more advanced cases of cholesterol granuloma, where the lesion has no epithelial lining, the primary goal of treatment is drainage of the lesion and restoration of aeration. Unfortunately, cholesterol granuloma recurs in up to 60% of cases, so MRI scans are thought to be the best approach for long- term follow-up after surgery [2,4].

A search of the literature revealed only three cases of familial hypercholesterolemia with bilateral cholesterol granuloma. The first case was a 35-year-old man with known familial hypercholesterolemia who had a destructive lesion in the left mastoid region, the second case was a 19-year-old patient in whom CT showed large cystic lesions bilaterally, and the third case was a 45-year-old woman who had cholesterol granuloma in the cerebrum. All three of these patients had familial hypercholesterolemia and were managed surgically. The second and third cases underwent coronary artery bypass graft surgery at an early age before resection of cholesterol granuloma [5,6]. There should be a high level of suspicion for cholesterol granuloma in patients with familial hypercholesterolemia if they present with temporal bone or intracranial masses.

## 5 Conclusion

Cholesterol granuloma is a benign lesion characterized histologically by the presence of giant cells. Diagnostic radiological studies are reliable. Management is individualized according to the site, size, and extent of the lesion. Surgical intervention is reserved for advanced cases.

## Funding

No source of funding.

## Ethical approval

IRB approval has been guaranteed by King Fahad Medical City. IRB Log number: 18-678.

## Consent

Written informed consent was obtained from the patients for publication of this case report and accompanying images. A copy of the written consent is available for review by the Editor-in-Chief of this journal on request.

## Author contribution

Nouf Albakheet has contributed in writing the introduction, case, and discussion.

Yazeed Al-shawi has contributed in writing the case.

Yasser Orz has contributed in writing the case.

Mohammed Bafaqeeh has contributed in writing the case.

Hanadi Fatani has contributed in writing the case.

Ibrahim Shami has contributed as the supervisor.

## Registration of research studies

researchregistry4999.

## Guarantor

Dr. Ibrahim Shami.

## Patient perspective

Patient perspective about the treatment has not been asked.

## Provenance and peer review

Not commissioned, externally peer-reviewed.

## Declaration of Competing Interest

No conflict of interest.
